# Generative adversarial network based digital stain conversion for generating RGB EVG stained image from hyperspectral H&E stained image

**DOI:** 10.1117/1.JBO.28.5.056501

**Published:** 2023-05-31

**Authors:** Tanwi Biswas, Hiroyuki Suzuki, Masahiro Ishikawa, Naoki Kobayashi, Takashi Obi

**Affiliations:** a Tokyo Institute of Technology, Department of Information and Communications Engineering, Tokyo, Japan; b Gunma University, Center for Mathematics and Data Science, Maebashi, Japan; c Saitama Medical University, Faculty of Health and Medical Care, Hidaka, Japan; d Tokyo Institute of Technology, Institute of Innovative Research, Tokyo, Japan

**Keywords:** generative adversarial network, digital stain conversion, H&E stained image, Verhoeff’s van Gieson stained image, hyperspectral imaging, image-to-image translation

## Abstract

**Significance:**

Quantification of elastic fiber in the tissue specimen is an important aspect of diagnosing different diseases. Though hematoxylin and eosin (H&E) staining is a routinely used and less expensive tissue staining technique, elastic and collagen fibers cannot be differentiated using it. So, in conventional pathology, special staining technique, such as Verhoeff’s van Gieson (EVG), is applied physically for this purpose. However, the procedure of EVG staining is very expensive and time-consuming.

**Aim:**

The goal of our study is to propose a deep-learning-based computerized method for the generation of RGB EVG stained tissue from hyperspectral H&E stained one to save the time and cost of conventional EVG staining procedure.

**Approach:**

H&E stained hyperspectral image and EVG stained RGB whole slide image of human pancreatic tissue have been leveraged for this experiment. CycleGAN-based deep learning model has been proposed for digital stain conversion while images from source and target domains are of different modalities (hyperspectral and RGB) with different channel dimensions. A set of three basis functions have been introduced for calculating one of the losses of the proposed method, which retains the relevant features of EVG stained image within the reduced channel dimension of the H&E stained one.

**Results:**

The experimental results showed that a set of three basis functions including linear discriminant function and transmittance spectrum of eosin and hematoxylin better retained the essential properties of the elastic fiber to be discriminated from collagen fiber within the reduced dimension of the hyperspectral H&E stained image. Also, only a smaller number of paired training data with our proposed training method contributed significantly to the generation of more realistic EVG stained image with more precise identification of elastic fiber.

**Conclusions:**

RGB EVG stained image is generated from hyperspectral H&E stained image for which our model has performed two types of image conversion simultaneously: hyperspectral to RGB and H&E to EVG. The experimental results show that the intentionally designed set of three basis functions contains more relevant information and prove the effectiveness of our proposed method in generating realistic RGB EVG stained image from hyperspectral H&E stained one.

## 1 Introduction

In histopathology, tissue sections that consist of thin layers with morphological features are observed under the microscope for disease diagnosis and prognosis. As unstained tissues are naturally of very low contrast and provide less information about the tissue components, tissue sections are stained with proper coloring reagent or dye so that complex tissue morphology can be observed efficiently and underlying function can be understood precisely. Hematoxylin and eosin (H&E) stain is the most common staining technique, which is applied routinely on almost all types of tissue specimens.^1^ H&E stain shows cytoplasm and fibers in pink color and nuclei in blue color.

Elastic fiber presenting in connective tissues of different body parts is responsible for the lifetime physiologic elasticity of the organ.^2^ Different studies show that abnormality in the concentration of the elastic fiber in the tissue is related to different types of diseases.^3^^–^^5^ Specially, in case of pancreatic ductal carcinoma (PDAC), vascular and ductal tissues are known to have specific intensity and frequency of the elastic fiber, which is a significant feature of diagnosing the disease.^6^

**Fig. 1:**
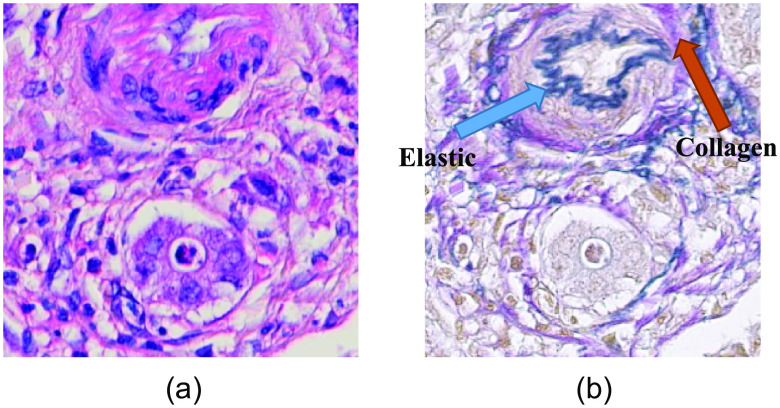
(a) H&E stained image with no distinguishable property of elastic and collagen fiber (b) EVG stained image of same tissue area with distinguishable information of elastic and collagen fiber.

Though H&E staining is the most commonly used staining technique, it cannot distinguish elastic and collagen fiber because of their similar color (pink) and pattern. In conventional pathology, special staining technique, such as Verhoeff’s van Gieson (EVG), is applied on spatially consecutive tissue where H&E has been applied previously. EVG stain shows elastic fiber in deep blue and collagen fiber in orchid color (Fig. 1), thus makes them easily distinguishable.^7^ However, procedure of EVG staining is very complex, time consuming, and costly. So, this study aims to generate EVG stained image in a less complex, time saving, and cost-efficient way. Moreover, as in conventional pathology, H&E and EVG staining are performed on two different consecutively aligned tissues; pathologists cannot observe the effect of different staining on same tissue specimen. However, this study provides the convenience of observing the effect of different stains on same tissue specimen.

The hyperspectral imaging (HSI) technology has been developed rapidly over the past decade. After having many successful applications of HSI in the arenas of agriculture,^8^^,^^9^ remote sensing,^10^ military purposes^11^ etc., it is being recently used in medical applications, such as macro-pathology^12^ and histopathology.^13^ In histopathology, along with the spatial information, hyperspectral images provide very precise and comprehensive spectral information of tissue components, which is not possible to obtain with RGB images. So, in this study, H&E stained hyperspectral images have been processed to obtain RGB EVG stained images virtually (or computationally). Deep learning based generative adversarial network (GAN) model has been deployed, which investigates the key morphology of H&E tissue from both of its spectral and spatial information, extracts the embedded properties of EVG tissue, and finally represents it with the characteristics of EVG stained image.

In recent years, different GAN based methods have been incorporated for image-to-image translation purposes of various histopathological applications, e.g., stain normalization^14^ where color variations within the same type of stained images introduced by different experimental factors are addressed by standardizing the color globally, virtual, or stainless staining^15^^,^^16^ where unstained images are converted to a specific type of stained images in a computerized way, and digital stain conversion^17^ where one type of stained images is transformed to another digitally. Some of these attempts involve images of which source and target domains are given by same modality; for example, RGB to RGB.^14^ Though some of the approaches adopt images of different modalities, the implementation of their proposed method is fully supervised and are highly dependent on the availability of the large number of prior aligned paired (images of same area that are exactly aligned and scale adjusted despite being obtained from two sources) training data. In computational pathology, previous studies have been found to perform digital stain conversion by obtaining a transformation function through matrix manipulation that maps certain tissue components of one domain to another.^18^^,^^19^ These approaches are not capable enough to analyze complex function of very high dimensional data. They are also unable to convert all the tissue components to its equivalent counterpart.

Septiana et al.^20^ have performed an experiment involving H&E and EVG stained images and have proposed a function termed as linear discriminant function (LDF) for distinguishing elastic and collagen fiber by applying linear discriminant analysis on H&E stained HSI.^20^ They have considered only the spectral information of H&E stained HSI disregarding the spatial information. Their proposed method is fully supervised requiring all paired training data from both domains, and the results provide only the information of elastic and collagen fibers ignoring all other tissue components.

To the best of our knowledge, this is the first work where hyperspectral H&E stained images have been leveraged to obtain their equivalent RGB EVG stained images in the pixel level. Here, the proposed method is converting two factors at the same time: hyperspectral to RGB and H&E to EVG. Our proposed method is designed to utilize the complete spectral information provided by the H&E HSI without reducing the spectral dimension. For doing so, we have faced challenges to calculate training losses and have tried to address the issue by considering a small number of basis function that best preserves the underlying important features of EVG stained image. Being inspired by the concept of transfer learning,^21^^,^^22^ our proposed method consists of two training phases. The first training phase contributes to the generation of realistic RGB EVG stained image from hyperspectral H&E stained image in a completely unsupervised way nullifying the necessity of paired training data. The second training phase is for refining the generated EVG stained image in supervised way with a small number of paired training data, which ensures that the finally obtained generated EVG stained is more realistic with less noise and more detailed information of elastic fiber. The combination of unsupervised and supervised training phases has alleviated the dependency of our proposed model on the availability of large number of exact paired training images, which is labor intensive and difficult to obtain. The detailed explanation of this technique will be discussed in the methodology section.

Briefly, our work involves below three main contributions.

1. This paper introduces a GAN based computerized technique for generating EVG stained image in a less complex and cost-efficient way that would have been required for conventional EVG staining.
2. The proposed model performs two types of conversions simultaneously: one is staining transformation, H&E to EVG, and another is hyperspectral to RGB.
3. The spectral information of H&E stained images have been utilized to the fullest while introducing a set of basis functions for calculating one of the losses of the proposed method, which retains the relevant features of EVG stained image within the reduced channel dimension of the H&E stained one.

## 2 Material and Method

### 2.1 Data Acquisition

Inspired by the previous research in Ref. 6 where it has been shown that abnormality of elastic fiber is related to PDAC and the previous study in Ref. 20, where the author has tried to discriminate elastic and collagen fiber from H&E stained HSI, the experiment of this paper has been performed deploying images of H&E and EVG stained tissues of human pancreas from TissueArray.Com, LLC,^23^ which used to be Biomax Inc. previously. All the tissues have been collected under Health Insurance Portability and Accountability Act of 1996 (HIPAA)-approved protocols, maintaining highest ethical standards with the donor being informed completely and with their consent.

Hyperspectral H&E stained images are obtained by a HSI system containing a hyperspectral camera of model NH3 by EBA JAPAN CO., Ltd., attaching an optical microscope BX-53 by Olympus Corp. and a white light emitting diode (LED) light source with it. The system captures the transmittance information of the tissue, which provides images of 151 bands for 350 to 1100 nm wavelength with 5 nm interval. The spatial size of the hyperspectral image is 752×480. By removing the redundant information of the tissue, we have used the hyperspectral image of 61 bands, which corresponds to the wavelength from 420 to 720 nm. The illumination spectrum of white LED also ranges from 350 to 1100 nm with 5 nm interval, whereas spectral information ranging from 420 to 720 nm have been considered for this experiment.

EVG stained images used for this study are RGB images. RGB EVG stained images are whole slide image (WSI), which is obtained with a WSI scanner by Hamamatsu photonics K.K. EVG stained images used in this experiment have similar structural information as H&E stained image because EVG staining has been applied after bleaching the same tissue where H&E staining was applied before.

### 2.2 Data Preprocessing

For removing the effect of different noise source and preparing for further analysis, the hyperspectral image is calibrated by^24^

$$I_{t}(\lambda )=\frac{I(\lambda {)}_{\text{raw}}/E_{\text{raw}}-I(\lambda {)}_{d}/E_{d}}{I(\lambda {)}_{w}/E_{w}-I(\lambda {)}_{d}/E_{d}},$$ (1)

where, $I(\lambda {)}_{\text{raw}}$, $I(\lambda {)}_{d}$, and $I(\lambda {)}_{w}$ are raw image pixel, dark image pixel with LED off, and white image pixel of the glass slide with LED on, respectively. $E_{\text{raw}}$, $E_{d}$, and $E_{w}$ are exposure value of the raw image, dark image, and white image, respectively.

**Fig. 2:**
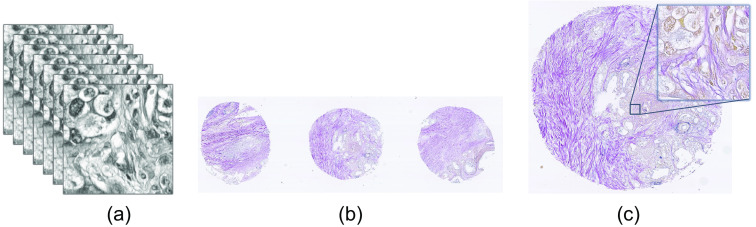
Description of experimental data (a) hyperspectral H&E stained image (b) RGB EVG stained WSI (c) same area as (a) cropped from EVG stained WSI.

**Fig. 3:**
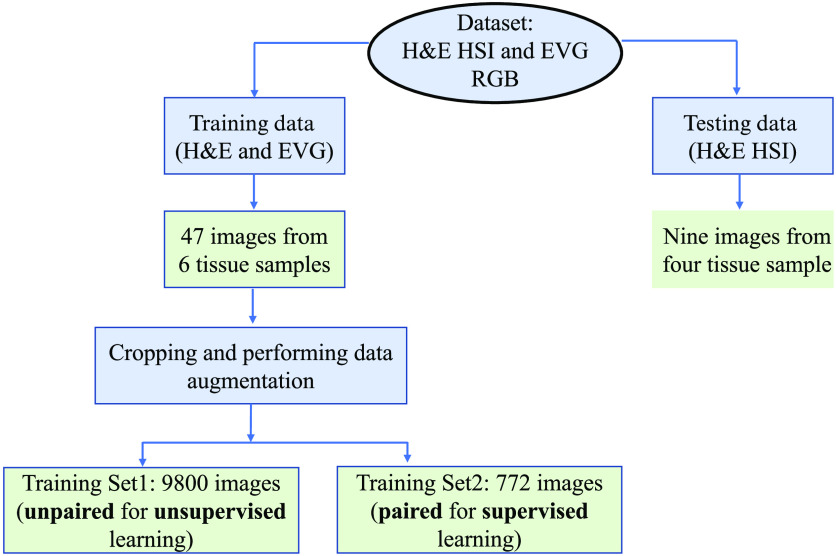
Graphical representation of experimental dataset.

Similar areas from the H&E stained HSI and EVG stained WSI have been cropped to prepare the training and test images, which is shown in Fig. 2. The dataset has been prepared prioritizing the parts of the tissue slides where both elastic and collagen fibers are present. Totally, nine H&E stained HSIs from four different tissue samples have been prepared for testing the model performance. The training dataset includes total 47 images from 6 different tissue samples. As our model has been trained in two different ways: unsupervised and supervised, there are two different corresponding training datasets. The unsupervised training dataset includes total 9800 unpaired (neither scale adjusted nor aligned exactly) images from each domain of H&E and EVG, which have been prepared after cropping the overlapped areas and performing augmentation using rotation and flipping. The spatial dimension of each training image is 128×128. The supervised training dataset includes 772 paired images, which are aligned and scale adjusted. For easier and clearer understanding, the experimental data setup has been illustrated in Fig. 3.

### 2.3 Preparing Ground Truth EVG Stained Image

To prepare training data for supervised learning and to determine the model performance, it is necessary to have the paired set of H&E stained and EVG stained image, which corresponds to each other. Since EVG stained and H&E stained images are captured with different cameras and in different time, it is hardly found that their structure is exactly aligned and scale adjusted. So, speeded-up robust features^25^ (SURF) feature based image registration has been performed between the H&E stained and the original EVG stained image cropped from WSI.

**Fig. 4:**
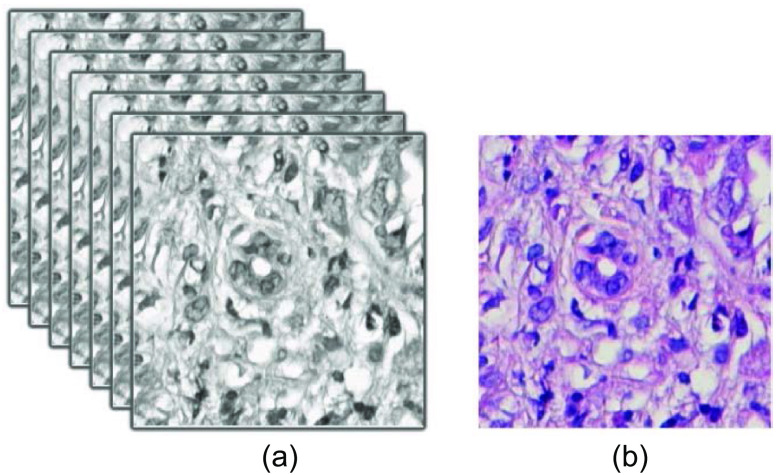
(a) Hyperspectral H&E stained image (b) converted sRGB H&E stained image.

**Fig. 5:**
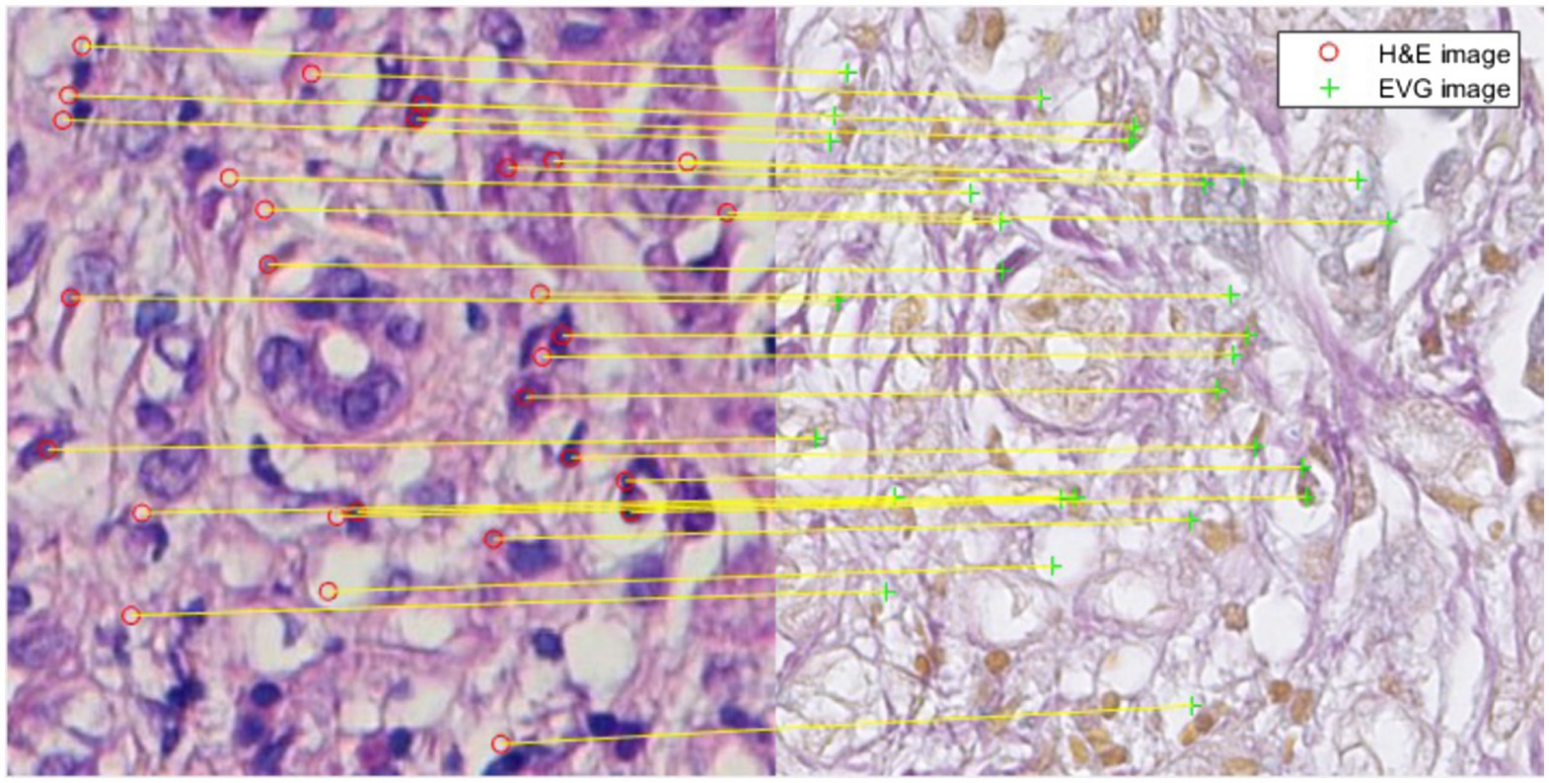
Matched SURF features between H&E stained (left) and EVG stained (right) images.

**Fig. 6:**
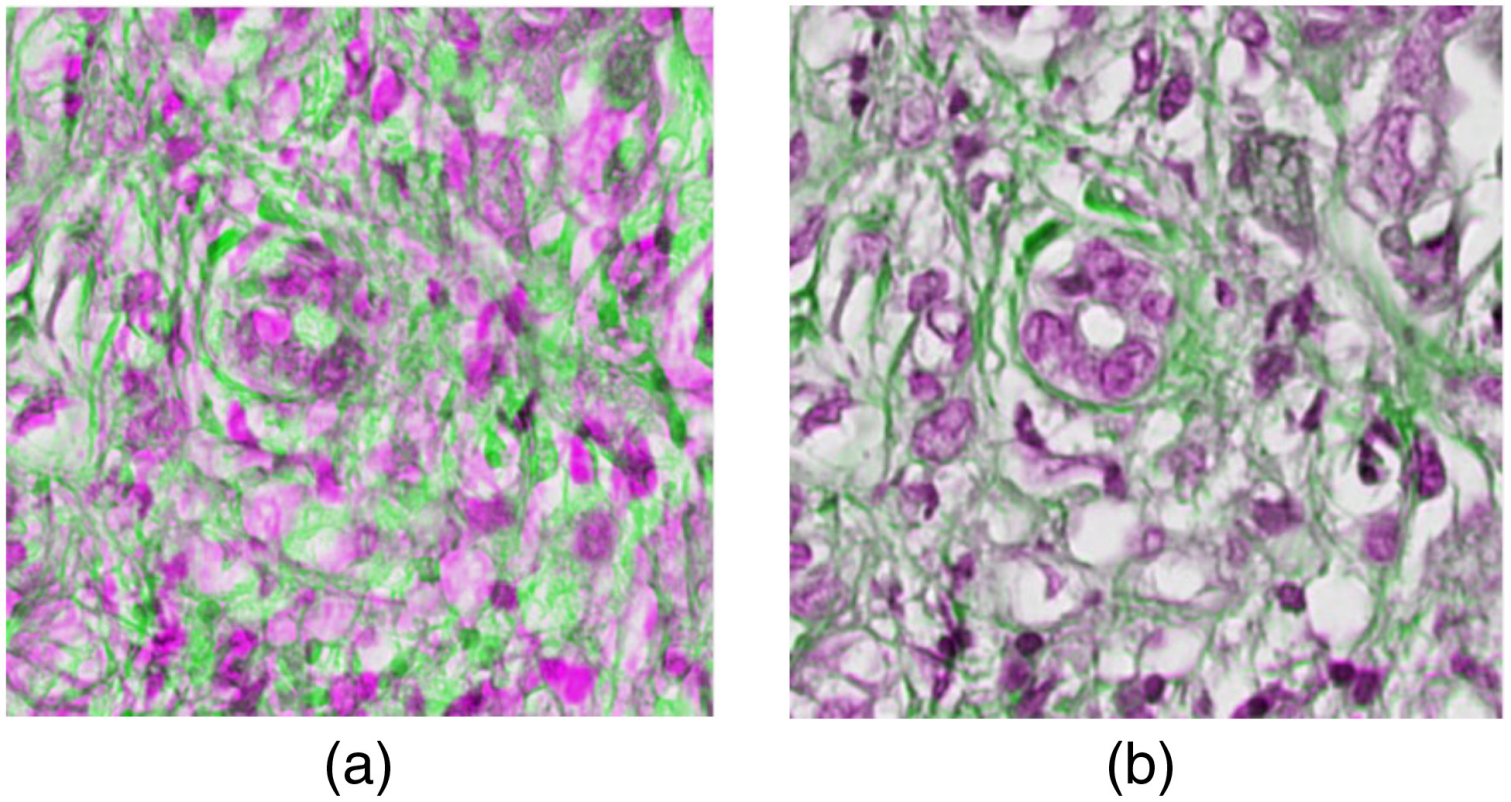
Overlapping view of H&E stained image (a) before registration (b) after registration.

To retain the simplicity and lower computational complexity of the registration method, hyperspectral H&E stained images have been converted to its corresponding standard RGB (sRGB) H&E stained images using color matching functions.^26^ Figure 4 represents an example of hyperspectral H&E stained image and its corresponding converted sRGB (which has a specific RGB color space with a limited color gamut developed to ensure standard color reproduction across different devices) H&E stained image. Due to the robustness, faster performance and scale and rotation invariant nature, hand crafted SURF^25^ feature has been detected and extracted both from H&E stained and EVG stained images (Fig. 5). SURF utilizes integral image and approximates Laplacian of Gaussian with box filter, which makes this algorithm faster and robust against scale. Feature matching has been performed by considering the distance between these two feature sets, and matched outliers are discarded applying M-estimator SAmple Consensus algorithm.^27^ Finally, applying the affine type of geometric transformation on the original EVG stained image, the registered EVG stained image is obtained. This registered EVG stained image is now scale adjusted and perfectly aligned with the original H&E stained image and has been leveraged as the ground truth image for this experiment. Figure 6 represents the overlapping view of original H&E and EVG stained image before (a) and after (b) performing the registration. The green color represents EVG stained image and magenta color represents H&E stained image when they are superimposed.

### 2.4 Methodology

**Fig. 7:**
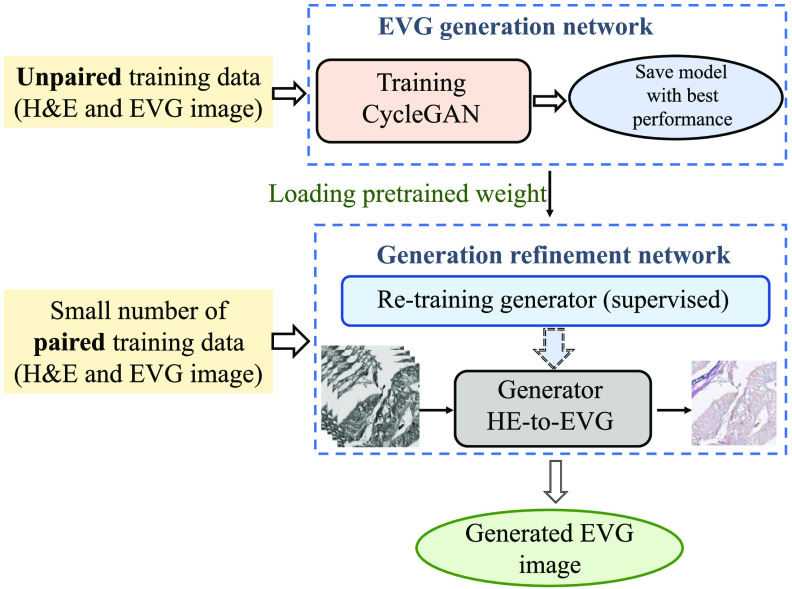
Workflow of proposed method for generating EVG stained from hyperspectral H&E stained image.

The proposed methodology consists of two parts: EVG generation network and generation refinement network, which have been designed with two different training phases (Fig. 7). The first part involves generation of realistic EVG stained image from hyperspectral H&E stained image leveraging CycleGAN architecture. The CycleGAN model is trained in an unsupervised way with unpaired dataset of hyperspectral H&E and RGB EVG stained images. The second part is called generation refinement network where the pre-trained generator that converts hyperspectral H&E stained images to their equivalent EVG stained images is re-trained in a supervised way with a small amount of paired data. The refinement network has been designed for refining the EVG stained image generated by the EVG generation network so that more realistic EVG stained image with reduced noise and more detailed identification of elastic fiber can be obtained. The detailed description of the proposed framework is mentioned in the next subsections.

### 2.5 EVG Generation Network

**Fig. 8:**
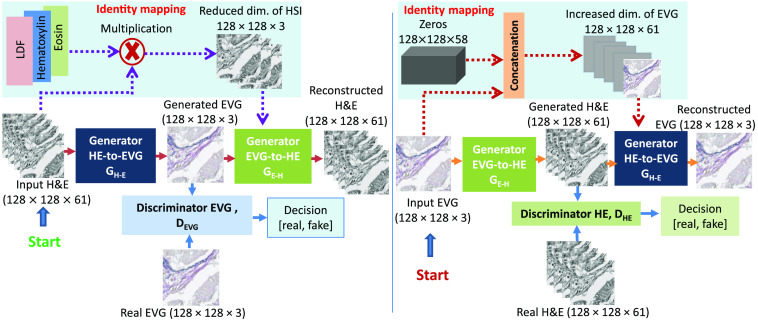
Proposed EVG generation network for generating RGB EVG stained image from H&E stained HSI.

In the EVG generation network, for two sets of training images H&E and EVG, which are unlabeled and unpaired, the modified CycleGAN model learns to map H&E stained image to EVG stained image and vice-versa. Like the CycleGAN model,^28^ our network consists of two generators $G_{H\text{-}E}$, which converts H&E HSI to RGB EVG, and $G_{E\text{-}H}$, which converts RGB EVG to H&E his, and two discriminators $D_{\mathrm{HE}}$ and $D_{\mathrm{EVG}}$, which discriminates real and fake H&E and EVG images, respectively. Unlike CycleGAN, the two generators and two discriminators are designed with different configurations to adopt our input images from different modalities. The methodology of the EVG generation network is shown in Fig. 8.

**Fig. 9:**
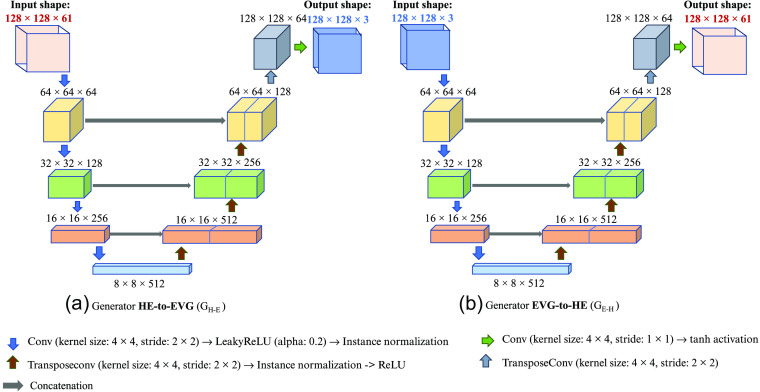
Architecture of two generators with varying input and output size: (a) generator HE-to-EVG (G_H-E_) and (b) generator EVG-to-HE (G_E-H_).

**Fig. 10:**
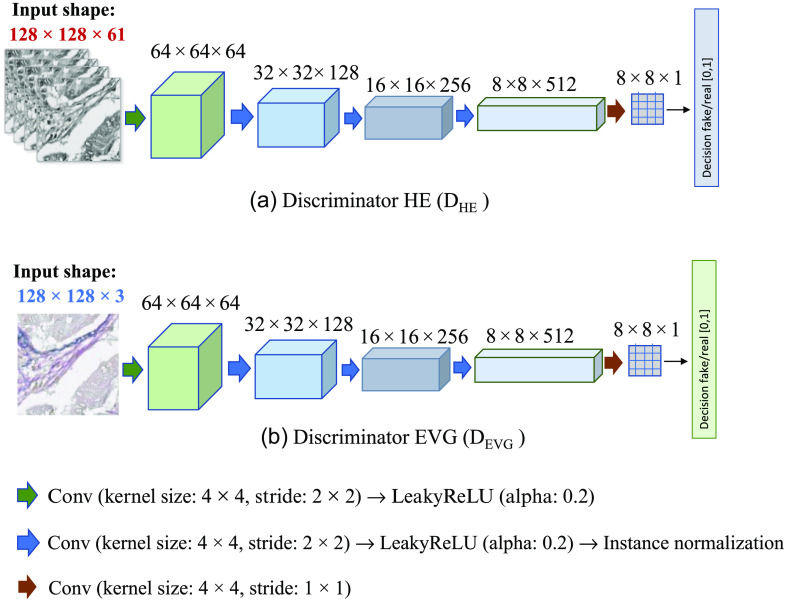
Architecture of the two discriminators with varying input size: (a) discriminator HE (D_HE_) and (b) discriminator EVG (D_EVG_).

Here, the modified CycleGAN model contains two heterogeneous generator architectures with different input dimensions and filter sizes. As our training images are of two different channel dimensions, the generator $G_{H\text{-}E}$ has been designed to take input image with channel dimension 61 and to produce the output image with channel dimension 3, whereas the generator $G_{E\text{-}H}$ has been designed to take input image with channel dimension 3 and to generate the output image with channel dimension 61. However, both are designed following the U-Net architecture^29^ with same spatial input and output dimension. Figure 9 illustrates the architecture of the two generators $G_{H\text{-}E}$ and $G_{E\text{-}H}$ from where it can be observed that the input and output image shapes of the two generators are different from each other. On the other hand, both discriminators used in this experiment are based on Patch-GAN^30^ architecture with different input channel dimensions to adopt two domains of images with different modalities. Figure 10 describes the configuration of these two discriminators $D_{\mathrm{HE}}$ and $D_{\mathrm{EVG}}$. Figure 10 shows that the input channel dimension of discriminator $D_{\mathrm{HE}}$ is 61 and the input channel dimension of discriminator $D_{\mathrm{EVG}}$ is 3 while both have same spatial dimension of 128×128.

### 2.6 Losses of EVG Generation Network

Like the original CycleGAN model,^28^ we have also considered three types of losses to train our EVG generation network: adversarial loss, cycle consistency loss, and identity loss. The total loss $L_{\text{Total}}$ is obtained by combining these three losses as

$$L_{\text{Total}}=L_{\text{adversarial}}+\lambda L_{\text{cycle}-\text{consistency}}+\gamma L_{\text{identity}},$$ (2)

where, $L_{\text{adversarial}}$ is adversarial loss, $L_{\text{cycle}-\text{consistency}}$ is cycle consistency loss multiplied by a scalar value λ, and $L_{\text{identity}}$ is identity loss multiplied by another scalar value. Adversarial loss and cycle consistency loss are calculated in the same way as Ref. 28. The method we have used to calculate identity loss has been described in next subsection.

### 2.7 Identity Loss

We expect that while translating images, the generators do not change images unnecessarily, and they translate image only when they should. This is done by reversing the inputs of the two generators and it is expected to have the output same as its original domain. This is called identity mapping and the loss calculated is called identity loss, which can be represented by

$$L_{\text{identity}}=E_{\mathrm{HE}∼p_{\text{data}}(\mathrm{HE})}(\|G_{E-H}({\mathrm{HE}}_{\text{Red}})-{\mathrm{HE}}_{\mathrm{Org}}\|)+E_{\mathrm{EVG}∼p_{\text{data}}(\mathrm{EVG})}(\|G_{H-E}({\mathrm{EVG}}_{\mathrm{Inc}})-{\mathrm{EVG}}_{\mathrm{Org}}\|).$$ (3)

For calculating the identity loss, we need to use 61 channel H&E stained HSI as the input of generator $G_{E\text{-}H}$, which is originally designed to take input image of channel 3, and need to use EVG stained image of channel 3 as the input of generator $G_{H\text{-}E}$, which is originally designed to take input image of channel 61. To solve this problem, a 61-channel EVG image (${\mathrm{EVG}}_{\mathrm{Inc}}$) must be generated from the original EVG stained image (${\mathrm{EVG}}_{\mathrm{Org}}$) and a 3-channel H&E image (${\mathrm{HE}}_{\text{Red}}$) must be generated from the original H&E HSI (${\mathrm{HE}}_{\mathrm{Org}}$). We have concatenated 58 channel zeros of same spatial dimension to the EVG stained image to make it of 61 channels, which is represented as ${\mathrm{EVG}}_{\mathrm{Inc}}$ in Eq. 3. The reduced dimensional H&E has been represented as ${\mathrm{HE}}_{\text{Red}}$ in Eq. 3. The task of reducing the dimension of H&E stained HSI to 3 is challenging as we have to retain all the detailed spectral information in these 3 channels which was used to be carried out by 61 channels previously. We have adopted several approaches to prepare ${\mathrm{HE}}_{\text{Red}}$, which are described below.

#### 2.7.1 Choosing three channels of H&E HSI

We have chosen several sets, 3 channels out of 61 channels of the H&E stained HSI. Empirically, it has been found that for the set of channels 10, 11, and 12 corresponding to the wavelength 465, 470, and 475 nm provides better conversion result in terms of differentiating elastic and collagen.

#### 2.7.2 Using LDF and principal components

**Fig. 11:**
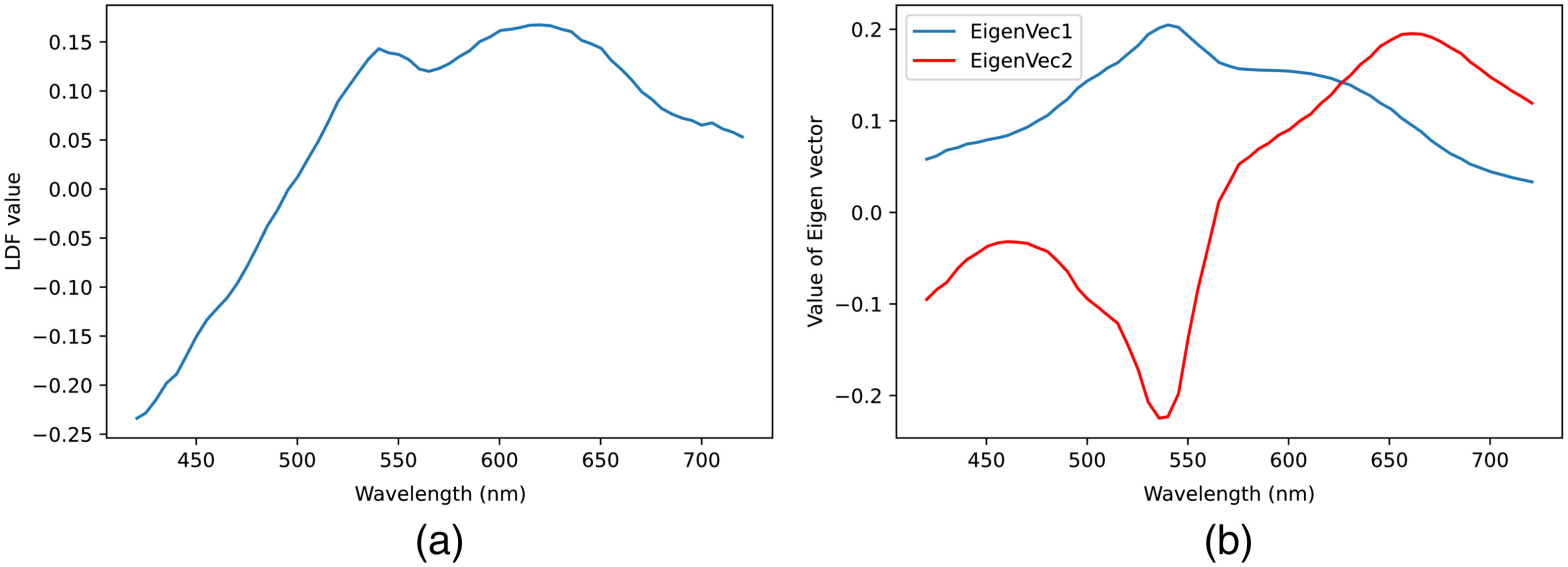
(a) Spectrum of LDF and (b) eigenvectors obtained from H&E stained HSI.

As using principal component analysis (PCA) is a well-recognized approach of dimension reduction and has been proved to be efficient in case of reducing the dimension of hyperspectral pathological images,^15^^,^^31^ we have also considered applying PCA for reducing the channel dimension of hyperspectral H&E stained image from 61 to 3. By investigating the linear combination of wavelengths, PCA optimizes the pixel values retaining maximum variations in the least-square sense. Empirically, it has been found that first two components together correspond to the variance over 95% and considering the third component does not add any significant variation. So, we concatenated the first two principal components (PCs) and LDF multiplied by H&E HSI to represent the H&E HSI with reduced dimension. This LDF was designed in Ref. 20 to classify the spectrum of elastic and collagen fibers from H&E HSIs [Fig. 11(a)], and we expect that the LDF helps to enhance the accuracy of the color conversion in the elastic and collagen fiber regions, which is important in diagnostic of PDAC. The Eigen vectors of the first two PCs have been represented in Fig. 11(b) where horizontal axis represents the wavelengths of the H&E HSI and the vertical axis represents the value of the Eigen vectors.

#### 2.7.3 Using LDF, Hematoxylin and Eosin spectrum

**Fig. 12:**
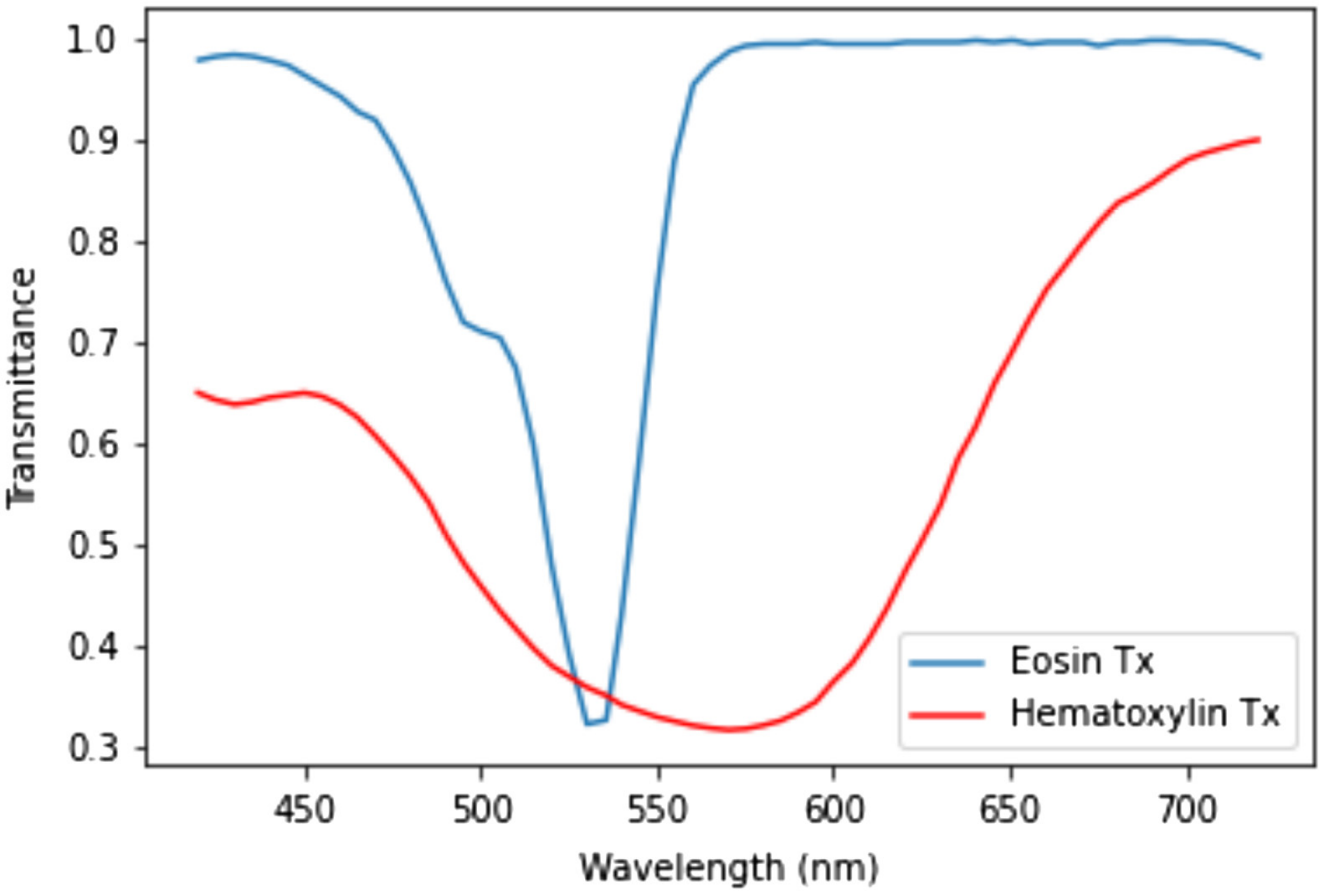
Eosin and hematoxylin transmittance spectrum.

We also have employed intentionally designed basis functions to reduce the dimension of HSI. The first one is LDF, which is previously mentioned in Sec. 2.7.2. The other two basis functions are the transmittance spectrum of eosin and hematoxylin.^32^ Since H&E stained tissues are mainly composed of eosin and hematoxylin, we expect that the two spectrums help to catch the color information of H&E stained tissues comprehensively. As our image contains the transmittance information of the tissue, from the relation of transmittance (T) and absorbance (A) shown as

$$A=-\log_{10}\;T,$$ (4)

we have obtained the transmittance spectrum of hematoxylin and eosin from their absorbance spectrum,^32^ which is shown in Fig. 12. We have prepared a set of three vectors including the LDF and the hematoxylin and eosin transmittance spectrum and have performed matrix multiplication with the hyperspectral H&E stained image. Thus, a tensor of dimension 128×128×3 has been found for each his, which has been used for calculating identity loss.

### 2.8 Training Details

Our proposed method consists of two different training phases. In the first training phase, the CycleGAN model of the EVG generation network is trained with unpaired training dataset. Here, the adversarial loss has been measured in terms of mean square error (MSE) and cycle consistency and identity loss have been measured using mean absolute error. We have used Adam for the optimization purpose with learning rate 0.0002. Empirically, choosing the value of λ and γ as 5.0 and 0.5, respectively, has been found to provide better result. We have set the batch size varying from 1 to 6 with no noticeable change in the experimental result. The final model has been chosen after 26 epochs and it takes around 35 min for each epoch to complete.

The second training phase involves the generation refinement network where the generator $G_{H\text{-}E}$ with best performance is chosen from the previous stage. The generator is then re-trained in a supervised way with a small number of paired images. The MSE loss between the generated and ground truth image is considered to re-train the model. Adam optimizer with a reduced learning rate of 0.00003 has been considered for re-training.

The experiment is implemented using Keras with TensorFlow backend and run on the GPU Quadro RTX 6000.

## 3 Results

We have performed both the qualitative and quantitative evaluation of the generated EVG stained images. Section 3.1 summarizes the results of the EVG generation network where we have tried to find out the best possible way to leverage CycleGAN when images from two domains are of different modalities, such as hyperspectral and RGB in our case. Section 3.2 represents the result of generation refinement network where we have tried to show the effectiveness of the two training phases of our proposed method.

### 3.1 Results of EVG Generation Network

**Fig. 13:**
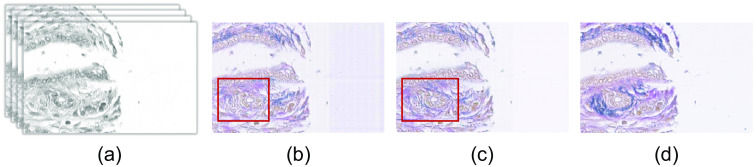
Demonstrating the effectiveness of considering identity loss. (a) Input H&E stained HSI. Generated EVG stained image (b) without considering identity loss (c) considering identity loss (using LDF, eosin and hematoxylin) (d) ground truth EVG stained image.

At first, we have evaluated the qualitative result of generated EVG stained image with the EVG generation network. Figure 13 represents the comparative result of the EVG generation network with and without considering identity loss. From the resultant images, it can be observed that EVG stained images considering identity loss is less noisy than that of without identity loss approach. Also, the performance of identifying elastic fiber (blue color) is better in case of considering identity loss, which is clearly visible inside the rectangle marked with red color.

**Fig. 14:**
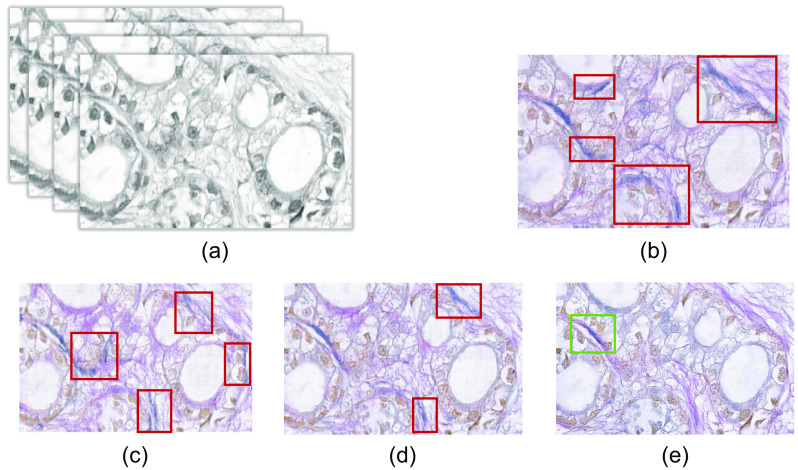
Demonstrating that the use of LDF, hematoxylin and eosin spectrum provide better performance. (a) Input H&E stained HSI. Output EVG stained image with (b) channels 10, 11, and 12 (c) considering LDF and PCs (d) considering LDF, hematoxylin and eosin spectrum for identity mapping. (e) Ground truth EVG stained image.

Next, we have compared the result of considering different approaches of identity mapping as mentioned in the Secs. 2.7.1–2.7.3. Figure 14 represents the result of generated EVG stained images while considering channels 10, 11, and 12, combination of LDF and PCs, and combination of LDF, eosin and hematoxylin spectrum for identity loss calculation. Visual comparison of the generated EVG stained image with the ground truth EVG stained image represents that except the elastic fiber parts, all other tissue components of the H&E stained image have been adapted appropriately to their corresponding EVG stained domain. There are some areas where elastic fiber has been generated falsely, which have been marked with red rectangle in the resultant images [Figs. 14(b)–14(d)]. The region containing the elastic fiber in the ground truth EVG stained image has been marked with green rectangle in Fig. 14(e). Comparing with other identity mapping approaches, generated EVG stained image with considering LDF, eosin and hematoxylin spectrum for identity mapping contains less amount of falsely generated elastic fiber. The generated EVG stained images are also less noisy here comparing to other identity mapping approaches.

**Fig. 15:**
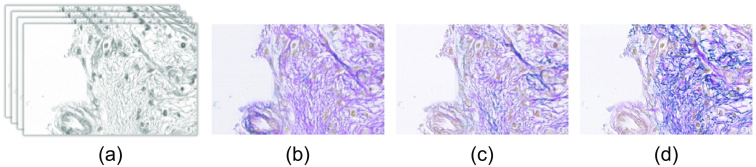
Demonstrating the effectiveness of using hyperspectral H&E over RGB H&E as input image for generating RGB EVG stained image: (a) input H&E stained image, (b) output EVG stained image from RGB H&E, (c) output EVG from hyperspectral H&E, and (d) ground truth EVG stained image.

We have also compared the cases of generating EVG stained image from Hyperspectral H&E and from its corresponding sRGB H&E, and the result has been represented in Fig. 15. Here, we can observe that the performance of mapping elastic and collagen fiber along with other tissue components is much better for EVG stained image generated from hyperspectral H&E stained image than that of sRGB H&E stained image. Moreover, the model trained with sRGB H&E stained images has been converged after 59 epochs, whereas the model trained with H&E hyperspectral image has converged only after 26 epochs. So, comparing to H&E stained HSI, model with RGB H&E stained image is requiring lots of more training iterations to converge.

For the quantitative evaluation of the generated EVG stained image with the ground truth EVG stained image, the metrics structural similarity index (SSIM), peak signal to noise ratio (PSNR) and root MSE (RMSE) have been deployed. For both output and ground truth images being RGB, RMSE, PSNR, and SSIM can be calculated as follows.

RMSR and PSNR both have very close relationship to MSE, which calculates the pixel wise error between images. MSE between the ground truth EVG stained image (${\mathrm{EVG}}_{\mathrm{GT}}$) and output EVG stained image (${\mathrm{EVG}}_{\text{out}}$) can be calculated as

$$\mathrm{MSE}=\frac{1}{H\times W\times C}\overset{H}{\underset{x=1}{\sum }}\overset{W}{\underset{y=1}{\sum }}\overset{C}{\underset{z=1}{\sum }}[{\left({\mathrm{EVG}}_{\mathrm{GT}(x,y,z)}-{\mathrm{EVG}}_{\text{out}(x,y,z)}\right)}^{2}],$$ (5)

where H, W, and C represents height, width, and number of channels, respectively. ${\mathrm{EVG}}_{\mathrm{GT}(x,y,z)}$ is the pixel value of the ground truth EVG stained image at the x, y coordinate of channel z and ${\mathrm{EVG}}_{\text{out}(x,y,z)}$ represents the pixel value of output EVG stained image at x, y coordinate of z channel. RMSE can be obtained from the MSE value as

$$\mathrm{RMSE}=\sqrt{\mathrm{MSE}}.$$ (6)

PSNR is useful to determine the level of noise present in the image and can be calculated as

$$\mathrm{PSNR}=10\;\log_{10}(\frac{\max^{2}}{\mathrm{MSE}}).$$ (7)

Here, the value of max is 255.

SSIM is another useful metric, which evaluates the structural similarity between images. SSIM is calculated considering three different components, such as luminance, contrast, and structure. The final SSIM is the multiplicative combination of these three terms. SSIM of ground truth EVG $\left({\mathrm{EVG}}_{\mathrm{GT}}\right)$ and output EVG (${\mathrm{EVG}}_{\text{out}}$) for a single channel can be defined as^33^

$$\mathrm{SSIM}({\mathrm{EVG}}_{\mathrm{GT}},{\mathrm{EVG}}_{\text{out}})={\left[l({\mathrm{EVG}}_{\mathrm{GT}},{\mathrm{EVG}}_{\text{out}})\right]}^{\alpha }.{\left[C({\mathrm{EVG}}_{\mathrm{GT}},{\mathrm{EVG}}_{\text{out}})\right]}^{\beta }.{\left[S({\mathrm{EVG}}_{\mathrm{GT}},{\mathrm{EVG}}_{\text{out}})\right]}^{\gamma }$$ (8)

where, α>0, β>0, and γ>0 are used to adjust the importance of these three components. We have considered the value of α=β=γ=1

$$l({\mathrm{EVG}}_{\mathrm{GT}},{\mathrm{EVG}}_{\text{out}})=\frac{2\mu_{{\mathrm{EVG}}_{\mathrm{GT}}}\mu_{{\mathrm{EVG}}_{\text{out}}}+C_{1}}{\mu_{{\mathrm{EVG}}_{\mathrm{GT}}}^{2}+\mu_{{\mathrm{EVG}}_{\text{out}}}^{2}+C_{1}},$$ (9)

$$C({\mathrm{EVG}}_{\mathrm{GT}},{\mathrm{EVG}}_{\text{out}})=\frac{2\sigma_{{\mathrm{EVG}}_{\mathrm{GT}}}\sigma_{{\mathrm{EVG}}_{\text{out}}}+C_{2}}{\sigma_{{\mathrm{EVG}}_{\mathrm{GT}}}^{2}+\sigma_{{\mathrm{EVG}}_{\text{out}}}^{2}+C_{2}},$$ (10)

$$S({\mathrm{EVG}}_{\mathrm{GT}},{\mathrm{EVG}}_{\text{out}})=\;\frac{\sigma_{{\mathrm{EVG}}_{\mathrm{GT}}{\mathrm{EVG}}_{\text{out}}}+C_{3}}{\sigma_{{\mathrm{EVG}}_{\mathrm{GT}}}\sigma_{{\mathrm{EVG}}_{\text{out}}}+C_{3}},$$ (11)

where $l({\mathrm{EVG}}_{\mathrm{GT}},{\mathrm{EVG}}_{\text{out}})$ compares luminance of ${\mathrm{EVG}}_{\mathrm{GT}}$ and ${\mathrm{EVG}}_{\text{out}}$. $C({\mathrm{EVG}}_{\mathrm{GT}},{\mathrm{EVG}}_{\text{output}})$ compares the contrast of ${\mathrm{EVG}}_{\mathrm{GT}}$ and ${\mathrm{EVG}}_{\text{out}}$ and $S({\mathrm{EVG}}_{\mathrm{GT}},{\mathrm{EVG}}_{\text{output}})$ compares structure of ${\mathrm{EVG}}_{\mathrm{GT}}$ and ${\mathrm{EVG}}_{\text{out}}$. $\mu_{{\mathrm{EVG}}_{\mathrm{GT}}}$, $\mu_{{\mathrm{EVG}}_{\text{out}}}$, $\sigma_{{\mathrm{EVG}}_{\mathrm{GT}}}$, $\sigma_{{\mathrm{EVG}}_{\text{out}}}$, and $\sigma_{{\mathrm{EVG}}_{\mathrm{GT}}{\mathrm{EVG}}_{\text{out}}}$ represents the mean, standard deviations, and cross-covariance of ${\mathrm{EVG}}_{\mathrm{GT}}$ and ${\mathrm{EVG}}_{\text{out}}$. $C_{1}$, $C_{2}$, and $C_{3}$ are regularization constant for luminance, contrast, and structure terms where

$$C_{1}={\left(0.01*L\right)}^{2},\;C_{2}={\left(0.03*L\right)}^{2},\;\text{and }C_{3}=C_{2}/2.$$

The value of L is 255 for our data. Considering the above equations, the SSIM of Eq. 8 is simplified to

$$\mathrm{SSIM}({\mathrm{EVG}}_{\mathrm{GT}},{\mathrm{EVG}}_{\text{out}})=\frac{\left(2\mu_{{\mathrm{EVG}}_{\mathrm{GT}}}\mu_{{\mathrm{EVG}}_{\text{out}}}+C_{1})(2\sigma_{{\mathrm{EVG}}_{\mathrm{GT}}{\mathrm{EVG}}_{\text{out}}}+C_{2}\right)}{\left(\mu_{{\mathrm{EVG}}_{\mathrm{GT}}}^{2}+\mu_{{\mathrm{EVG}}_{\text{out}}}^{2}+C_{1})(\sigma_{{\mathrm{EVG}}_{\mathrm{GT}}}^{2}+\sigma_{{\mathrm{EVG}}_{\text{out}}}^{2}+C_{2}\right)}.$$ (12)

As both our output and ground truth EVG stained images are RGB, SSIM has been calculated for each R, G, and B channel separately, and the final SSIM value has been obtained by averaging the SSIM values of these three channels, which is represented as

$${\mathrm{SSIM}}_{\mathrm{RGB}}=\frac{1}{\mathrm{Ch}}\overset{\mathrm{Ch}}{\underset{i=1}{\sum }}\mathrm{SSIM}({\mathrm{EVG}}_{{\mathrm{GT}}_{i}},{\mathrm{EVG}}_{{\text{out}}_{i}}),$$ (13)

where Ch is the number of channels, ${\mathrm{EVG}}_{{\mathrm{GT}}_{i}}$ is ground truth EVG image of i’th channel and ${\mathrm{EVG}}_{{\text{out}}_{i}}$ is output EVG image of i’th channel. The maximum possible value of SSIM is 1.

Higher SSIM and PSNR value represents better quality while lower value of RMSE represents better quality. Table 1 summarizes our experimental results in terms of these quality metrics of the generated EVG stained images by averaging the result of all the test images. The third column of the table represents the RMSE of whole images, whereas fourth column of the table represents the RMSE for only the fibrous regions of the images. To extract the fibrous regions, the EVG stained images have been converted to their corresponding Hue saturation value (HSV) color space from where the color information of elastic and collagen fibers has been obtained. The binary mask of this fibrous region has been prepared with this color information and have been leveraged to extract the fibrous regions from the ground truth and generated EVG stained images. As our fiber regions remain in the color range of deep blue to pink, for extracting the fibrous regions we used two separate color range of HSV space: one is for blue to pink with lower limit [110, 50, 50] and upper limit [170, 255, 255], and another is for different variation of black with lower limit [120, 0, 55] and upper limit [150, 30, 90]. The first row of Table 1 represents the method where identity loss is not considered and the second row represents the result of using sRGB H&E stained image for generating EVG stained image. The third to fifth rows represent the result of different approaches of identity mapping, such as considering channel 10, 11, and 12 of hyperspectral H&E stained image, combination of LDF and PCs, and combination of LDF, eosin and hematoxylin spectrum, respectively. From Table 1, we can see that generating EVG stained image from hyperspectral H&E stained image with considering the combination of LDF, eosin and hematoxylin spectrum for identity mapping has outperformed all other approaches.

**Table 1:** Quantitative evaluation of the generated EVG stained image of EVG generation network. Values are bold to highlight better result.

Methods	SSIM	PSNR	RMSE (whole image)	RMSE (fibrous regions only)
Without identity loss	0.6827	22.4431	20.3562	18.3058
sRGB H&E	0.6999	22.0285	21.2376	18.5495
Channels 10, 11, and 12 for identity mapping	0.6670	21.4501	22.2768	17.7645
LDF and PCs for identity mapping	0.6812	22.5402	20.1754	**17.5510**
LDF, eosin and hematoxylin for identity mapping	**0.7065**	**22.7108**	**19.6684**	17.5838

### 3.2 Results of Generation Refinement Network

**Fig. 16:**
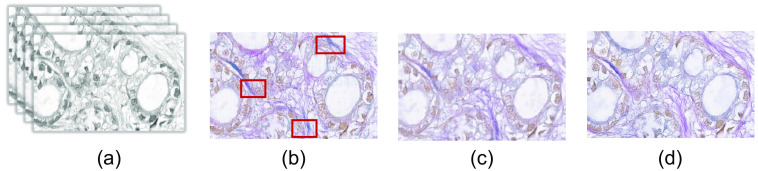
Illustrating the effectiveness of retraining (supervised) generator in reducing falsely generated elastic fiber: (a) input H&E stained HSI, (b) generated EVG before re-training, (c) generated EVG after re-training, and (d) ground truth EVG stained image.

From the experimental result of the above subsection, it has been observed that our proposed CycleGAN based EVG generation network generates better quality RGB EVG stained image from Hyperspectral H&E stained image while considering LDF, eosin and hematoxylin spectrum for calculating the identity loss. However, the generated EVG stained images still contain some falsely generated elastic fiber. To generate more realistic EVG stained image with mitigated effect of falsely generated elastic fiber, the CycleGAN model with LDF, eosin and hematoxylin spectrum for identity mapping has been considered and re-trained in a supervised way in the generation refinement part of our proposed method. Figure 16 represents the comparative view of the generated EVG stained images before and after retraining the generator $G_{H\text{-}E}$. It is clearly visible that the generation refinement network has successfully removed the falsely generated elastic fiber and has contributed to the generation of more realistic EVG stained image.

**Fig. 17:**
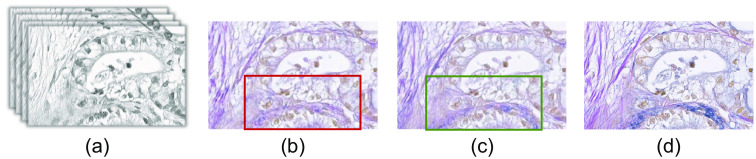
Demonstrating that the use of pre-trained CycleGAN weight improves performance. (a) Input H&E stained HSI. Generated EVG stained image (b) without and (c) with pretrained CycleGAN weight. (d) Ground truth EVG stained image.

In our experiment, we have also investigated the effect of the pre-trained weight that comes from the CycleGAN based EVG generation network, the first phase in Fig. 7. Figure 17 shows the generated EVG stained image with and without considering the pre-training weight of the generator $G_{H\text{-}E}$ obtained from the EVG generation network. From the generated EVG stained images, we can see that without considering the pre-trained weight, the generator model cannot have the information of the elastic fiber and the generated EVG stained images includes no or very less information of elastic fiber.

Table 2 represents the quantitative evaluation of the generated EVG stained image from generation refinement network. The first row represents the result before re-training with the supervised way. The second row represents the result after training the generator $G_{H\text{-}E}$ with the supervised way without considering the pre-trained CycleGAN weight. The last row represents the result of generation refinement network after re-training the generator $G_{H\text{-}E}$ with considering the pre-trained CycleGAN weight. From the table, we can see that when the generator model is initialized with the pre-trained CycleGAN weight, retraining it with a small number of paired data has improved the quality of the generated EVG stained image significantly. Though there is small difference between the cases with and without considering pre-trained CycleGAN weight, the information of elastic fiber cannot be obtained from the generated EVG stained image where pre-trained CycleGAN weight is not leveraged.

**Table 2:** Quantitative evaluation of the generated EVG stained image of generation refinement network. Values are bold to highlight better result.

Methods	SSIM	PSNR	RMSE (whole image)	RMSE (fibrous regions only)
LDF, eosin and hematoxylin for identity mapping	0.7065	22.7108	19.6684	17.5838
Without pretrained weight of CycleGAN	0.7461	24.2819	16.8106	14.8191
Generation refinement with pretrained weight	**0.7547**	**24.7831**	**15.5535**	**12.1340**

## 4 Discussion

This study proposes a deep learning based computerized method for generating RGB EVG stained image from hyperspectral H&E stained image. Our approach investigates the feasibility of generating RGB EVG stained image in a GAN based unsupervised way to alleviate the labor-intensive work of preparing paired training data. The resultant images show that though all other parts of the tissue components have mapped very closely to the original EVG from H&E, the proper identification of elastic fiber only with unsupervised way is still challenging. So, to improve the quality of the generated EVG stained image and to mitigate the effort of preparing large number of paired data, this study utilizes a small number of paired data to re-train the generator model of the CycleGAN to generate more refined EVG stained image. Both from the qualitative and quantitative result, we can see that re-training the generator model has improved the generated EVG stained image quality significantly with less noise and more precise identification of elastic fiber. The unsupervised CycleGAN model is contributing to discriminate elastic and collagen fiber, whereas the supervised re-training method is contributing to the generation of more refined, less noisy realistic EVG stained image. There is less difference in the quality metrices between the approaches without and with considering the pre-trained weight of the CycleGAN based EVG generation network. The reason behind it is that most of the test images contains very small amount of elastic fiber and when we consider the whole image for comparing the quality, it is being difficult to observe the effect. So, when we compare RMSE of only fibrous regions, there is noticeable difference between these two approaches and it is clear that considering the pre-trained generator weight is contributing to the generation of better quality EVG stained image with more precise identification of elastic fiber.

As this study is designed to utilize the complete spectral information provided by the H&E HSI as the input of the deep learning model, it takes very longer time to complete training and an explicit identity mapping technique was required to design. One potential solution to this problem may be reduction of the dimension of HSI first and to utilize the reduced dimensional data as the input information for stain conversion model. Next step of this study includes reducing the dimension of the H&E HSI so that maximum information can be retained within this reduced dimensional data while performing stain conversion efficiently with faster speed and much higher accuracy. This experiment has been conducted within our limited scope of data availability. For the practical implementation of the proposed method, more training data from different sources including patient variability and environmental effect needs to be considered for improved robustness.

## 5 Conclusion

In this paper, we have presented a computerized way of digital stain conversion utilizing the unpaired image-to-image translation technique of GAN. The generated RGB EVG stained images have been obtained from hyperspectral H&E stained images for which our model has performed two types of image conversion at the same time: hyperspectral to RGB and H&E to EVG. In addition, for solving the problem of calculating identity loss while using images of different modalities for image-to-image translation purpose, we have found the best set of functions that successfully reduces the channel dimension of the hyperspectral H&E stained images while the embedded property of EVG stained images is best retained. The additional supervised training method has alleviated the dependency on large amount paired training data and has provided significant improvement in the quality of the generated EVG stained image comparing to the approach where only the unsupervised training phases is adopted. The resultant images also show that the performance of distinguishing elastic and collagen fiber in the generated EVG stained image is much higher for hyperspectral H&E stained image than RGB H&E stained image. Both the qualitative and quantitative results support the effectiveness of the proposed method for generating realistic EVG stained image from hyperspectral H&E stained one.

## Code, Data, and Materials Availability

Because of privacy policy for medical information, data utilized in this experiment are not publicly available at this moment. However, the code, dataset, and other materials of this research may be obtained from the authors upon reasonable request.
